# Headache at the emergency room: Etiologies, diagnostic usefulness of the ICHD 3 criteria, red and green flags

**DOI:** 10.1371/journal.pone.0208728

**Published:** 2019-01-07

**Authors:** Joe Munoz-Ceron, Varinia Marin-Careaga, Laura Peña, Jorge Mutis, Gloria Ortiz

**Affiliations:** 1 Neurology Department, Universidad del Rosario-Hospital MEDERI, Bogotá, Colombia; 2 Neurology Department, Fundación Universitaria Sanitas–Clínica Universitaria Colombia, Bogotá, Colombia; 3 Sociedad Boliviana de Neurología, La Paz, Bolivia; 4 Fundación Cardio Infantil, Bogotá, Colombia; University of Würzburg, GERMANY

## Abstract

**Introduction:**

Non-traumatic headaches account for 0.5 to 4.5% at the emergency department (ED). Although primary headaches represent the most common causes, the likelihood of ominous etiology has to be considered by clinicians in order to avoid diagnostic and therapeutic pitfalls. Due to the absence of biological or imaging findings to diagnose primary headaches we hypothesize ICHD 3(International Headache criteria 3) criteria as a useful tool at the moment to identify and to establish a difference between those patients who are undergoing primary headaches and those who will need advanced diagnostic strategies.

**Objectives:**

To determine the usefulness of ICHD 3 criteria to differentiate primary from non-primary headaches at the emergency department (ED).

**Methods:**

During five weeks all the patients complaining of headache attended at the triage unit at the ED were interviewed, examined and classified as having primary or non-primary headaches by means of ICHD 3 criteria. Those patients with primary headaches were treated according to standard of care protocols and followed up by means of phone call communication after 48 hours to assure satisfactory outcome. Those patients classified as having non-primary headaches (secondary headaches and neuralgias) were admitted for additional diagnostic and therapeutic interventions. Between both groups we compared the prevalence of fulfilled criteria for primary headaches and the proportion of traditional red flags such as age, sleep headache onset, associated symptoms, abnormal neurological exam, sudden onset, and nonresponse to analgesics in addition to previous consultation before this evaluation.

**Results:**

Headache was responsible for 244 (2.3%) out of 10450 admissions at the ED, 77.8% were females. Primary, non-primary (secondary plus neuralgias) and unclassified headaches were 59.4%, 32% and 8.6% respectively. Migraine and cervical myofascial pain were the most frequent etiologies for primary and non-primary causes respectively. Factors associated to non-primary etiologies were immunosuppression (OR: 2.7 IC 95% 2.3–3.3) and age older than 50 (OR: 2.7 IC 95% 2.01–3.62). Abnormal neurological exam, sudden and sleep headache onset were not statistically significant.

Factors found to be associated with primary headaches were: fulfilling ICHD 3 criteria (OR: 18.7, IC95% 7.1–48.6), history of migraine (OR: 2.9 IC 95% 2.1–3.9), and history of similar episodes (OR: 2.7 IC 95% 2.3–3.3).

**Conclusion:**

This data suggests that fulfilling ICHD 3 criteria could be useful to differentiate primary from non-primary headaches. This observation is also valid for immunosuppression, age older than 50, history of migraine and history of similar episodes.

## Introduction

Neurological conditions account for 10 to 15% [1] of patients visiting the emergency department (ED), of which 13 to 27.8% are represented by patients with non-traumatic headache as the main complaint [2][3]. These numbers, according to several descriptions represent 0.5 to 2.8% of the emergency visits[4]. Although this complaint seems to be infrequent, it represents a diagnostic challenge due to the wide variability of presenting signs and symptoms and the peculiarity of the ED setting marked by issues related to time and proper use of diagnostic resources. In order to determine which patients need further studies, clinical practitioners and guidelines have traditionally employed the presence or absence of red flags [5], nevertheless it could be possible to face patients who in spite of having a secondary etiology, present none of these traditional red flags (age over 50, immunosuppression, abnormal neurological exam, or associated symptoms). This argument has motivated us to look for different strategies that could be useful to establish what patients will need further studies and what patients will need only symptomatic relief based on the origin of headache, primary or non-primary etiologies. This strategy also includes the search for factors that indicate primary origin (green flags) which, according to our knowledge, have not been described in the emergency service. The International Headache Society recommends using the International Headache (ICHD3) to classify and diagnose these disorders. This tool is based on the number and duration of episodes, accompanying symptoms and exclusion of differential diagnosis [6]. This tool has good applicability in terms of reliability[7][8], sensitivity and prediction of migraine when assessed in clinical research [9][10]

We have hypothesized that in addition to traditional red flags, the International Headache Criteria 3B [11] along with green flags could be useful tools to achieve this goal. In this study we aimed to determine the usefulness of the ICHD 3 criteria to differentiate primary vs non-primary headaches along with the determination of the main etiologies and the value of red and green flags to determine primary vs non-primary etiologies.

## Methods

During seven weeks, from 7 am to 7 pm, we enrolled all the patients who visited the emergency room-triage area with headache as their chief complaint. The study was conducted at a level IV hospital, in in the city of Bogota, Colombia. Patient inclusion was conducted in January and February, 2017. We excluded patients with history of cranial trauma during the last three months, pregnant women and individuals younger than eighteen. All the patients were interviewed in order to obtain clinical information with respect to duration, number of episodes, pain type, associated symptoms, alleviating and aggravating factors and medical history in order to determine variables included in ICHD 3. An expert neurologist performed a physical exam emphasizing on neurological exploration. According to the ICHD 3 classification, patients were classified as presenting primary or non-primary (secondary and neuralgias) headaches, this was considered the initial diagnosis. Written informed consent was obtained according to international recommendations. Those patients with a primary headache diagnosis were treated according to institutional protocols and were followed up as outpatients by means of phone communication after 48 hours to assure improvement of symptoms. The group with non-primary headache diagnosis was followed up in the hospital for further evaluation and treatment in order to determine the precise etiology of the headache. In cases where there was suspicion of structural lesions a brain MRI plus contrast was conducted, including MRA and MRV. If these studies did not show any findings, or if the suspicion included inflammatory or infectious causes a cerebrospinal fluid study was performed as well. The end of the follow up process was established by the pain resolution in the primary group and the determination of diagnosis in the subjects with non-primary etiologies, this was considered the definitive diagnosis. Afterward, we compared the prevalence of associated factors in order to determine the likelihood of primary vs non-primary headaches, these factors were age over 50, onset characteristics and associated symptoms, history of immunological disorders, history of migraine, tension type headache or similar episodes to the current pain. We also compared the prevalence of any analgesic treatment before, previous consultation and fulfilling ICHD 3 criteria for primary headaches between both groups al at the time of the initial diagnosis. This study was approved by the institutional ethics committee of the Rosario University.

Analyses were performed using STATA 14. Descriptive statistic was reported by mean of frequencies; association measures were showed using relative risks, confidence intervals and p values, which were considered significant when they were lower than 0.05.

## Results

The incidence of non-traumatic headache was 2.2%, 244 cases in 11,280 visits during seven weeks. Middle-aged women were the most affected group. Most of the patients visited the hospital for the first time during the current episode (Table 1). Eleven patients were lost to follow-up.

**Table 1:** Demographic characteristics.

Variable	
Age	
Female	37.8(IQR25-75%)24-53
Male	40.2(IQR25-75%)18-49
Gender	
Female	77.8%
Male	21.2%
Headache duration (hours)	
Female	105.6(IQR25-75%)16-296
Male	296.3(IQR25-75%)20-326
Analgesics use before consultation	
Female	83.5%
Male	85.5%
First Visit in the current episode/Subsequent-visits	72/28%

IQR: Interquartile range

Regarding etiologies, primary headaches were the most frequent causes followed by secondary etiologies and neuralgias respectively. Out of all the cases 8.6% could not be classified according to the ICHD 3. Migraine including status migrainosus accounted for the main etiologies in the primary group. Tension-type headache (TTH) and trigeminal autonomic cephalalgias (TACs) were infrequent etiologies in this study, 1.3 and 5% respectively. In the group of Non-primary headaches (secondary and neuralgias) the three main conditions were cervical myofascial pain, neuralgias/painful neuropathies and headache associated to systemic infections (Table 2).

**Table 2:** Etiologies according to ICHD 3 classification.

Group n(%)	Etiology	n
Primary Headaches 145 (59.4)	1.1 Migraine without aura	34
	1.2 Migraine with aura	73
	1.4.1 Status migrainosus	29
	1.4.2 Persistent aura without infarction	1
	2. Tension type headache	2
	3.1.1 Cluster Headache	2
	3.2.1 Paroxysmal hemicrania	1
	3.3.1 SUNCT	1
	3.4 Hemicrania Continua	1
	4.7.1 Probable primary stabbing headache	1
Secondary Headaches 63(25.9)*	6.2.1 Headache attributed to non-traumatic intracerebral hemorrhage	3
	6.2.2 Headache attributed to non-traumatic subarachnoid hemorrhage (SAH)	2
	6.3.2 Headache attributed to arteriovenous malformation (AVM)	1
	7.1.1 Headache attributed to idiopathic intracranial hypertension (IIH)	1
	7.4.1 Headache attributed to intracranial neoplasm	4
	9.1.3.1 Acute headache attributed to intracranial fungal or other parasitic infection	2
	9.2.2 Headache attributed to systemic viral infection	7
	10.3.2 Headache attributed to hypertensive crisis without hypertensive encephalopathy	6
	11.4/11.5 Headache attributed to disorders of the ears and paranasal sinuses	12
	11.7 Headache attributed to temporomandibular disorder	1
	12.1 Headache attributed to somatization disorder	1
Non-classified 21(8.6)	14.1 Headache not elsewhere classified	21
Painful cranial neuropathies and other facial pains 15(6.1)	13.4 Occipital neuralgia/other extracranial neuralgias	8
	13.5 Optic neuritis	1
Appendix	A11,2.5- Headache attributed to cervical myofascial pain	23

*Including 23 cases of A11,2.5- Headache attributed to cervical myofascial pain

Regarding risk, we found that 82% of the cases were non-life-threatening conditions (migraine, tension type headache, cervical myofascial, neuralgias/painful neuropathies, temporomandibular joint disorder) and the correspondent number were conditions that can result in death (central nervous system infections, systemic infections, intracranial neoplasms and vascular disorders).

Patients over 50, along with those who had immunological disorders were more likely to have secondary etiologies. We did not find statistical significance for sudden onset headache, onset at sleep, abnormal neurological exam and associated symptoms (diplopia, vertigo and syncope). Patients who fulfilled the ICHD 3 criteria in addition to those individuals who had a history of migraine and similar headache episodes were more likely to undergo primary etiologies (Tables 3 and 4).

**Table 3:** Factors associated with for secondary etiologies.

Variable	PR	CI 95%	p
Immunosuppression	2.7	2.3–3.3	0.03
Age over 50	2.7	2.0–3.6	0.02
Sudden onset	1.3	0.7–1.9	NS
Onset at sleep	1.1	0.4–1.8	NS
Abnormal Neuro exam	1.6	0.8–2.4	NS
Associated Symptoms (Syncope, vertigo, Diplopia)	0.8	0.1–1.5	NS

CI:Confidence interval, NS: Non significant

**Table 4:** Factors associated with primary Headaches.

Variable	PR	CI 95%	p
History of Migraine	2.9	2.1–3.9	0.03
History of similar episodes	2.7	2.3–3.3	0.02
Fulfilling ICHD 3 B criteria	18.7	7.1–30.3	<0.001

CI: Confidence Interval

## Discussion

This study found an incidence of non-traumatic headache of 2.3% at the ED, this finding is similar to reports which in spite of using different methodologies have described incidences between 0.6 to 2.8%[12,13,4,14,15], this headache percentage could be understood as a small number meaning that although headache is common in the general population seldom motivate patients to look for medical assistance at the emergencies services. Regarding the types of headache, primary etiologies accounted for the higher proportion of patients (58%), these results are somehow similar to those reported by Locker et al who reported that 81.2% of the patients were classified as part of this group [14]. However, some investigations (16) describe a higher proportion of patients with secondary headache. This finding probably explained by the source of information obtained from referrals to the neurology service, increasing the possibility to diagnose secondary etiologies.

With regards to the specific etiologies for primary headaches, migraine was the main condition in this study, representing 93.7% (migraine with aura, migraine without aura and status migrainosus) of the cases and 55% of the whole group. This finding suggests that although migraine is not a condition related to mortality, it has a high chance to generate disability during attacks, leading patients to the emergency service for symptomatic control.

In our study 1.3% of the patients fulfilled criteria for tension type headache. This low proportion could be understood as well taking into account the argument related to disability, which is expected to be low in patients with this type of headache reducing the likelihood to visit the emergency service. This result disagrees with studies [16,17] in which tension type headache was more prevalent than migraine. These observations could be explained by the fact of not using the ICHD criteria, or because the data was obtained when there were no clear differences between patients with cervical pain and tension type headache[13]. The low proportion of patients with trigeminal autonomic cephalalgias and group IV headaches is explained by their low prevalence in the general population.

With regards to non-primary etiologies, Headache attributed to cervical myofascial pain was the most frequent diagnosis 29.5% of this group and 9% of the total. This result represents an interesting finding which highlights the importance of correctly applying ICHD in order to avoid misdiagnosing patients with occipital and neck pain along with myofascial tenderness as tension type headache patients when criteria of duration a number of episodes are not fulfilled. Likewise, this finding could encourage considering reasons to include this diagnosis in the secondary headaches rather than in the appendix of the ICHD 3[11]. This analysis could be also valid for painful cranial neuropathies and other facial pains, 5.6% of the total in this study. In the ICHD 3 this group includes only entities that affect trigeminal, glossopharyngeal and occipital nerves, not considering other cranial nerves that when affected could lead to misdiagnosis of migraine due to due its severity, lateralized pain, and association to nausea and vomit.

Regarding the risk level, we found that 82% of patients underwent non-life threatening conditions, this result is similar to the 86.6% and 88% reported by Locker et al, and Ang et al respectively [12,18].

In order to determine predictive factors of etiology our study showed that age over 50 and history immunosuppression were associated to non-primary headaches, while history of similar episodes, history of migraine and fulfilling the ICHD 3 criteria were predictive of primary etiologies. Our results coincide with the observations of Locker et al [12] who described more risk of secondary etiologies in patients over 50. Nevertheless, there were discrepancies with abnormal neurological exam, sudden onset and associated symptoms, these variables were not statically significant in our analysis. To explain these differences, we consider the fact that in our study physical exam was conducted by a neurologist with focus in headache patients, increasing the probability to detect abnormalities such as trigeminal sings in patients with migraine and TACs, and subtle paresis in hemiplegic migraine. Regarding the sudden onset the disagreement could be attributed to the definition used which was less than five minutes in our study, and the fact that some of the migraines in our patients started during sleep. As for the associated symptoms, all of them non- significant, we included in our analysis the presence of vertigo, syncope, diplopia, excluding fever. We hypothesize that the association measures could have been significant if this variable had been included. Nevertheless, from the clinical point of view it is straightforward to consider a non-primary etiology when high temperature is documented.

In the context of predictors of primary etiologies, we found that fulfilling the ICHD 3B criteria showed the highest value to diagnose a primary headache. We hypothesize in this section that fulfilling items A and B (number and duration of episodes) represent the higher proportion of attributable risk to determine the diagnostic of a primary headache and that conversely to what have been described in outpatients [19] symptoms such as nausea, vomit, photo and phonophobia along with pain intensity are not useful in the emergency department to establish a difference due to the high likelihood to find this group of symptoms in headache syndromes independently of the etiology. Based on this finding we suggest that in addition to specific clinical situations, such as worst headache, recurrent headaches, progressive worsening headache [20], age, and immunological state, classifying patients according the presence or absence or ICHD 3 criteria for primary headaches of high prevalence could be useful at the moment to determine what patients belong to common primary etiologies and what cases need additional work up to determine a non-primary origin.

Although we consider that collecting information directly from triage service and the fact that all patients were analyzed by a head expert represent the main strengths of this study, our research has some limitations: first the follow up was done by means of telephonic contact without a direct clinical evaluation which was performed only in cases of re-consultation; second, those patients who consulted between 7:00 P.M. and 7:00 A.M. were not included, this factor could have modified the proportion of etiologies increasing the number of patients who required additional work up.

In conclusion, we consider that besides the traditional red flags and clinical scenarios the ICHD should be added to the tools to differentiate primary from non-primary headaches. Nevertheless, it is necessary to calculate association measures for each item of the diagnostic criteria included in the ICHD3 to obtain additional information.
